# Influence of ischemic core muscle fibers on surface depolarization potentials in superfused cardiac tissue preparations: a simulation study

**DOI:** 10.1007/s11517-012-0880-1

**Published:** 2012-03-13

**Authors:** Fernando O. Campos, Anton J. Prassl, Gunnar Seemann, Rodrigo Weber dos Santos, Gernot Plank, Ernst Hofer

**Affiliations:** 1 Institute of Biophysics, Medical University of Graz, Harrachgasse 21/IV, 8010 Graz, Austria; 2 Oxford e-Research Centre, University of Oxford, Oxford, UK; 3 Institute of Biomedical Engineering, Karlsruhe Institute of Technology, Karlsruhe, Germany; 4 Department of Computer Science and the Graduate Program in Computational Modeling, Federal University of Juiz de Fora, Juiz de Fora, Brazil

**Keywords:** Cardiac electrophysiology, Computer simulations, Extracellular potentials, Myocardial ischemia, Superfusion

## Abstract

Thin-walled cardiac tissue samples superfused with oxygenated solutions are widely used in experimental studies. However, due to decreased oxygen supply and insufficient wash out of waste products in the inner layers of such preparations, electrophysiological functions could be compromised. Although the cascade of events triggered by cutting off perfusion is well known, it remains unclear as to which degree electrophysiological function in viable surface layers is affected by pathological processes occurring in adjacent tissue. Using a 3D numerical bidomain model, we aim to quantify the impact of superfusion-induced heterogeneities occurring in the depth of the tissue on impulse propagation in superficial layers. Simulations demonstrated that both the pattern of activation as well as the distribution of extracellular potentials close to the surface remain essentially unchanged. This was true also for the electrophysiological properties of cells in the surface layer, where most relevant depolarization parameters varied by less than 5.5 %. The main observed effect on the surface was related to action potential duration that shortened noticeably by 53 % as hypoxia deteriorated. Despite the known limitations of such experimental methods, we conclude that superfusion is adequate for studying impulse propagation and depolarization whereas repolarization studies should consider the influence of pathological processes taking place at the core of tissue sample.

## Introduction

**Fig. 1:**
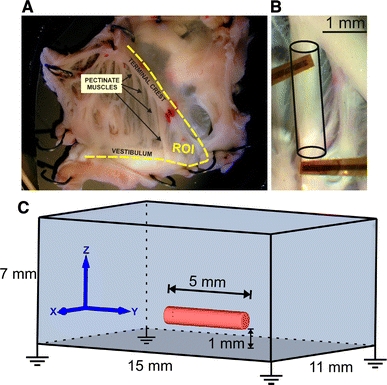
**a** Posterior part of the lower right atrium of a Rabbit heart (*ROI* region of interest) pinned on silicone rubber with tungsten needles and superfused with oxygenated Tyrode’s solution.** b** Ramification of the terminal crest into a pectinate muscle. Two four-electrode sensor arrays carried from flexible polyamide to measure extracellular potentials are shown.** c** Finite element model of a thin-walled cardiac sample, resembling the pectinate in** b**, immersed in a bath with four symmetrically arranged grounding electrodes

Multicellular cardiac preparations, such as isolated small animal atria (Fig. 1), pulmonary vein sleeves, ventricular slices as well as trabeculae and papillary muscles are widely used in experiments to study the physiology of cardiac function in health and disease [5, 21, 22, 40]. Unlike in isolated myocytes, gap junctions and extracellular matrix are preserved, allowing to characterize electrophysiological function under conditions similar to those in intact hearts (see Fig. 1b).

In contrast to larger preparations, where perfusion is essential to ensure adequate oxygen supply as well as wash out of waste products throughout the preparation [47], due to its simplicity, superfusion with oxygenated solutions is typically employed for thin-walled tissue samples [5, 17, 21–23, 40]. Although superfusion is adequate to maintain preparations viable for prolonged experimental procedures, this is not the case with larger samples where the thickness of the muscle wall exceeds the diffusion length [2]. In these cases, metabolic deficits build up with distance from superfused surfaces. High-energy phosphate stores in anoxic regions become depleted, hyperkalemia and acidosis develop due to insufficient wash out causing cells to become non-excitable and non-conductive.

Although these progressive changes which drive the formation of an ischemic [26, 32] and, ultimately, a myolytic core, clearly compromise integrated physiological functions such as active force generation [2], further elucidation is required to clarify as to which extent these pathological conditions in deeper layers of the tissue affect excitation spread and extracellular depolarization signals recorded close to the intact and viable surface. On one hand, the contribution of cells in ischemic regions as current sources during depolarization will be attenuated or even disappear [33]. On the other hand, these cells will influence electrophysiological behavior of viable cells in adjacent surface layers via electrotonic interactions [12]. A detailed quantitative understanding of these processes and their effects on excitation spread, as observed at the tissue surface, is key to allow a correct interpretation of experimental data recorded from such preparations.

In this study, we developed a biophysically detailed 3D model of a thin-walled cardiac tissue preparation (Fig. 1c) to investigate how superfusion-induced heterogeneities affect the recorded depolarization signals measured on the tissue surface. The model is based on the cardiac bidomain equations and considers the electrophysiological changes of inner muscle fibers under pathological conditions such as ischemia phase 1a, phase 1b and myolysis.

## Methods

### Governing equations

The set of bidomain equations describes the bioelectric activity in cardiac tissue [14]. The equations link intracellular and extracellular potential fields via transmembrane currents which act as sources:

1 $$ \nabla \cdot ({\varvec{\sigma}_{\rm i}} \nabla \phi_i) = \beta I_{\rm m}, $$

2 $$ \nabla \cdot ({\varvec{\sigma}_{{\rm e}}} \nabla \phi_{\rm e}) = -\beta I_{\rm m}, $$

3 $$ I_{\rm m} = C_{\rm m}\frac{\partial V_{\rm m} }{\partial t} + I_{\rm ion} (V_{\rm m},{\varvec{\eta}}) - I_{\rm stim}, $$

4 $$ \frac{\partial {\varvec{\eta}}}{\partial t} = {{\mathbf{g}}}(V_{\rm m},{\varvec{\eta}}), $$

5 $$ V_{\rm m} = \phi_{\rm i} - \phi_{\rm e}, $$

where ${\varvec{\sigma}}_{\rm i}$ and ${\varvec{\sigma}}_{\rm e}$ are the intracellular and extracellular conductivity tensors (respectively); β is the membrane surface to cell volume ratio; *I*_m_ is the transmembrane current density; *I*_stim_ is the stimulus current density; *C*_m_ is the membrane capacitance per unit area; *V*_m_ is the transmembrane voltage; and *I*_ion_ is the density of the total ionic current flowing through the membrane channels, pumps and exchangers, which in turn depends on *V*_m_ and on a set of state variables, ${\varvec{\eta}},$ which governs the dynamics of cellular processes such as gating or changes in concentrations within intracellular compartments. The vector-valued function ${\mathbf{g}}$ comprises the set of functions which describes the rate of change of the state variables ${\varvec{\eta}}.$ At the tissue boundaries, electrical isolation is assumed, which is accounted for by imposing no-flux boundary conditions on ϕ_i_.

In many experimental setups, including in vivo studies, cardiac tissue is surrounded by a conductive medium, such as blood or a superfusing Tyrode’s solution that can be modeled by an additional Poisson equation:

6 $$ \nabla \cdot (\sigma_{\rm b} \nabla \phi_{\rm e}) = 0, $$

where σ_b_ is the isotropic conductivity of the conductive medium. In this case, no-flux boundary conditions are assumed at the boundaries of the conductive medium, whereas continuity of the normal component of the extracellular current and continuity of ϕ_e_ are enforced at the tissue-bath interface. Typically, Dirichlet boundary conditions are applied in the extracellular domain to prescribe potentials as imposed by the electrodes of data acquisition systems, i.e., nodes in the mesh are chosen where ϕ_e_ is fixed either at zero or at a given time-varying stimulus voltage.

### Ischemic ventricular myocyte Model

The Mahajan–Shiferaw (MSH) model [24], which is represented by the terms $I_{\rm ion}, {\varvec{\eta}}$ and ${\mathbf{g}}(V_{\rm m},{\varvec{\eta}})$ in Eqs. 3–4, was used to describe cellular dynamics of normal rabbit ventricular myocytes. Following previous approaches, model modifications were introduced to represent the three main components of ischemia at the cellular level: hyperkalemia, acidosis and hypoxia [30, 39, 45]. Two distinct phases during the first hour after stopping perfusion can be distinguished [10], a first phase, referred to as phase 1a, which lasts for about 15 min and a second, later phase, referred to as phase 1b, lasting from 15 to 45 min. To account for the progression of ischemia, model parameters were adjusted to represent the combined effects of hyperkalemia, acidosis and hypoxia at 0 min (control); 5 and 10 min (phase 1a); 15 and 30 min (phase 1b) after stopping perfusion (see Table 1).

Hyperkalemia was introduced by increasing the extracellular potassium concentration [K^+^]_e_ [34, 39]. Since the MSH model does not explicitly account for pH value, effects of acidosis were modeled indirectly through a decrease in the L-type calcium and sodium channel conductances, *g*_Ca_ and *g*_Na_, respectively, and by shifting the voltage-dependent kinetics of the sodium current *V*_s,Na_ [3, 39, 45]. Hypoxia was mimicked by augmenting the MSH model with an adenosine triphosphate (ATP) sensitive potassium current, *I*_K,ATP_ [13], which activates when ATP concentration in the cytosol ([ATP]_i_) is reduced and the adenosine diphosphate concentration ([ADP]_i_) is increased. During ischemia phase 1b, changes in [K^+^]_e_ are moderate while acidosis and hypoxia worsen [30]. Cytosolic Na^+^ accumulates secondary to the inhibition of sodium–calcium exchanger (*I*_NaCa_) and the sodium–potassium pump (*I*_NaK_). In addition, ATP depletion and pH reduction reduce release current strength of ryanodine receptor channels (*g*_RyR_) as well as calcium uptake into the sarcoplasmic reticulum (ν_up_), [4, 6, 11, 25], which leads to cellular calcium overload. Metabolic inhibition is also known to augment the background calcium current, *I*_Ca,B_, and the calcium-sensitive nonselective cation current, *I*_ns,Ca_ [30]. However, since *I*_ns,Ca_ and *I*_Ca,B_ were not included in the MSH model formulation and considering that these currents are fairly small compared to the other currents, they were not taken into account. Parameter modifications to reflect phase 1b conditions were obtained based on the range of values during acute ischemia [13, 30, 39]. A summary of all parameters of cardiomyocytes under normal as well as ischemic conditions can be found in Table 1.

### Pseudo-1D model of an ischemic multicellular strand

A computer model of a 5-mm long strand of cardiac tissue with a diameter of 25 μm was developed to assess the impact of ischemia-related changes on impulse propagation at the tissue scale. The model geometry, which is close to 1D, was discretized at a spatial resolution of 25 μm using 3D hexahedral finite elements. Although 3D elements are employed, due to diameter which is small compared to the length of the preparation, the strand can be considered to behave like a 1D structure. During each simulation run, a particular condition, i.e., control, hyperkalemia, acidosis, hypoxia or ischemia at various stages, was assigned to the entire strand, where for each condition the MSH model was modified according to Table 1. In addition to cellular scale adaptations, progression of ischemia also induces a host of changes which affect electrophysiological properties at the tissue scale. Myocyte and vascular morphologies are altered, which, in turn, affect the conductivity ${\varvec{\sigma}}_{\rm e}$ of the extracellular matrix [20, 44]. Further, during ischemia phase 1b considerable dephosplorylation of Cx43 takes places due to prolonged acidification, leading to the progressive closure of gap junctions [6]. These changes are reflected in a reduced intracellular bidomain conductivity ${\varvec{\sigma}}_{\rm i}$ [3, 41]. Both ${\varvec{\sigma}}_{\rm i}$ and ${\varvec{\sigma}}_{\rm e}$ were further reduced to model the extreme case of irreversible cell damage, referred to as myolysis. Based on experimental measurements [7, 20], the relative changes in ${\varvec{\sigma}}_{\rm i}$ and ${\varvec{\sigma}}_{\rm e}$ for different stages of ischemia and myolysis are summarized in Table 2.

### 3D Model of a superfused thin-walled cardiac tissue

A finite element model of a thin-walled tissue preparation was developed to study the effects of ischemia-induced heterogeneities in the depth of the tissue on activation sequence, action potential (AP) morphology and extracellularly recorded depolarization signals. In this work, a geometry representative of cardiac muscle strips such as pectinate (as shown in Fig. 1b), trabecular and papillary muscles was chosen since these are widely to investigate the physiological functions of the heart [15, 21, 23]. In particular, rabbit papillary muscles have lengths that are between 3.5 and 5 mm long with a diameter of ∼1 mm [19, 42, 43]. Based on that, a cylindrical approximation of the muscle’s geometry of 5 mm length and 0.5 mm radius was generated (Fig. 1c). The model was composed of 35,498 vertices and 193,567 tetrahedral elements of 62.42 μm average edge length and a maximum aspect ratio of 4.14 [31]. The preferred axis of conduction was assumed to be aligned with the *y* axis, i.e., the main axis of the cylinder. The thin-walled muscle was immersed in a bath of dimensions 11 × 15 × 7 mm^3^ to account for bath-loading as it is always present with experimental set-ups employing superfusion [16, 36]. The muscle was positioned 1 mm above the bottom of the bath and centered in the *x–y* plane. 83,766 vertices and 464,891 tetrahedral elements of 185.02 μm average edge length and a maximum aspect ratio of 5.18 were used to discretize the bath.

**Fig. 2:**
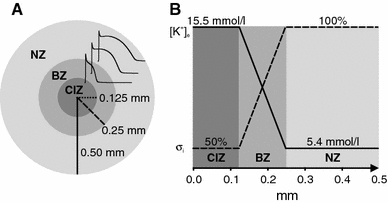
**a** Cross section of the 3D muscle preparation in Fig. 1C showing APs from central ischemic zone (*CIZ*), border zone (*BZ*) and normal zone (*NZ*).** b** Gradients of extracellular potassium concentration [K^+^]e and intracellular conductivity ${\varvec{\sigma}}_{\rm i}$ along the radial axis. Values for [K^+^]_e_ and ${\varvec{\sigma}}_{\rm i}$ in the CIZ and NZ are assigned according to ischemia phase 1b (30 min) and control, respectively (Tables 1, 2)

The assessment of transmural inhomogeneities of [K^+^]_e_, pH and myocardial energy metabolism in the heart during ischemia has been the focus of many experimental studies [8, 37, 46]. Accordingly, spatial heterogeneity secondary to the progression of ischemia was accounted for by assigning different sets of parameters to the central ischemic zone (CIZ), the border zone (BZ) [35] surrounding the CIZ and the normal zone (NZ). The depth of the NZ, within which superfusion is sufficient to avoid any impairment of physiological function, was chosen assuming a diffusion depth of 250 μm [1]. The remaining core of the muscle was subdivided into a BZ of 125 μm radius enclosing the CIZ (Fig. 2a). In the NZ, parameters were set according to control conditions to represent viable surface layers. In the CIZ, on the other hand, the same adjustments to cellular properties as well as ${\varvec{\sigma}}_{\rm e}$ and ${\varvec{\sigma}}_{\rm i}$ were made as before in the set-up of the 1D strand to account for different stages of ischemia. In the BZ, as depicted in Fig. 2b, parameters were linearly interpolated between NZ and CIZ [3, 18, 45].

Six tissue simulations were conducted to study the effects of progressive ischemia in the CIZ using the parameters detailed in Tables 1 and 2: control at 0 min with the entire cross section of the muscle intact; ischemic phase 1a (at 5 and 10 min); and phase 1b (at 15 and 30 min). At last, one simulation was conducted to assess a worst case scenario, i.e., the onset of irreversible cell damage in the whole core of the preparation: myolysis. In this extreme scenario, electrophysiological parameters in both CIZ and BZ were assigned according to phase 1b 30 min stage, but ${\varvec{\sigma}}_{\rm i}$ and ${\varvec{\sigma}}_{\rm e}$ were reduced to 10 and 50 %, respectively, of the values used in control.

**Table 1:** Model parameters for myocytes under normal and different stages of ischemia

Parameter	Stage of ischemia (min)				
	Control	Phase 1a		Phase 1b	
	0	5	10	15	30
Hyperkalemia					
[K^+^]_e _(mmol/l)	5.4	8.9	12.5	14	15.5
Acidosis					
* g*_Ca_ (%)	100	87.5	75	50	50
* g*_Na_ (%)	100	87.5	75	50	50
* V*_s,Na_ (mV)	0	1.7	3.4	3.4	3.4
* I*_NaCa_ (%)	100	100	100	60	20
Acidosis and hypoxia					
[Na^+^]_i_ (mmol/l)	14	14	14	17	20
* I*_NaK_ (%)	100	100	100	65	30
* g*_RyR_ (%)	100	100	100	50	5
ν_up_ (%)	100	100	100	95	90
Hypoxia					
[ATP]_i _(mmol/l)	6.8	5.7	4.6	3.5	2.0
[ADP]_i_ (μmol/l)	15	57	99	136	164

**Table 2:** Changes in extracellular (${\varvec{\sigma}}_{\rm e}$) and intracellular (${\varvec{\sigma}}_{\rm i}$) conductivities

Parameter (%)	Stage of ischemia (min)					
	Control	Phase 1a		Phase 1b		Myolysis
	0	5	10	15	30	>30
${\varvec{\sigma}}_{\rm e}$	100	75	65	60	55	50
${\varvec{\sigma}}_{\rm i}$	100	100	100	80	50	10

### Simulation protocols

The MSH model was solved by the Rush–Larsen method, with a time step of 10 μs. Initial conditions for all cell populations in the regions NZ, BZ and CIZ were computed for the instants 0, 5, 10, 15 and 30 min by pacing a single cell at a basic cycle length (BCL) of 500 ms (2 Hz) using cellular parameters as given in Table 1. Pacing was terminated when arriving at a stable limit cycle where differences in AP features between subsequent beats were small (<1 %, see Sect. 2.6 for further details about AP features). The state variables at the end of the protocol for each case were stored to be used as an input for the tissue simulations.

Bidomain parameters for the NZ were taken from the literature [29]: membrane capacitance *C*_m_ = 1 μF/cm^2^, surface-to-volume ratio β = 0.14 μm^−1^ and the conductivities σ_il_ = 0.174 S/m, σ_it_ = 0.019 S/m, σ_el_ = 0.625 S/m and σ_et_ = 0.236 S/m where the indices i and e indicate intracellular and extracellular, and l and t the longitudinal and transverse direction, respectively. The surrounding bath was considered isotropic with conductivity σ_b_ = 1 S/m.

**Fig. 3:**
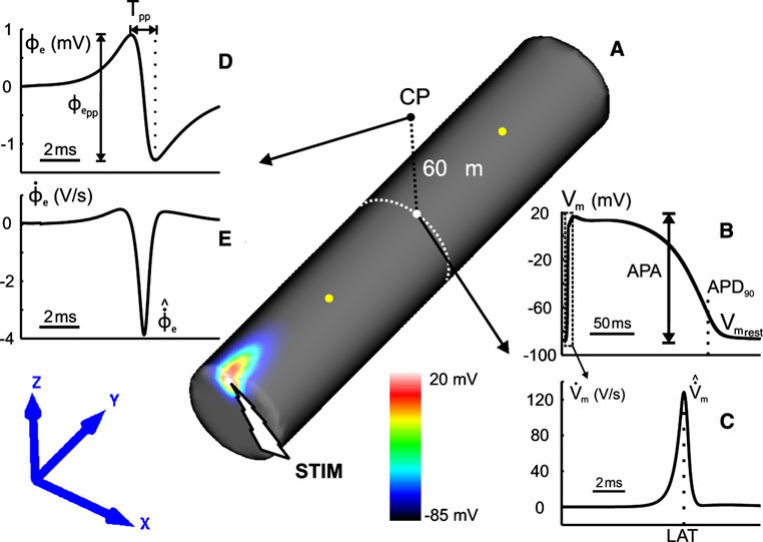
**a** Spatial distribution of *V*_m_ in the control model 1 ms after stimulation (*STIM*) at the* left top cusp*.* Yellow dots* indicate the location of the recording sites used to determine conduction velocity (CV).** b** Action potential at the surface of the NZ.** c** Time derivative of *V*_m_ during an AP.** d** ϕ_e_ signals at the observation point (*CP*) at 60 μm above the muscle’s surface.** e** Time derivative of ϕ_e_

In all tissue simulations, a transmembrane current stimulus was applied at the left top cusp of the muscle (Fig. 3a). Four symmetrically arranged grounding electrodes were placed at the lower corners of the bath (see Fig. 1c). To ensure steady-state at the tissue scale, the phase 1b set-up (which is the model with the most pronounced AP heterogeneity) was paced seven times at a BCL of 500 ms after initialization with the single-cell steady-state values. Limit cycle trajectories were verified by recording APs in all three regions NZ, BZ and CIZ within the central cross sectional plane of the muscle. Differences in AP morphologies (using metrics as described below, Sect. 2.6) between the last two paced beats at all three sites were negligible. After administering two stimuli, differences between two subsequent APs were <3 %. Therefore, for all other cases under study, only two pacing stimuli were delivered to arrive at a limit cycle.

### Data analysis

AP features were computed for all tissue simulations. APs were taken from the central cross-sectional area of the model (see Fig. 3) to avoid boundary artifacts. In the 3D case, APs from the CIZ were also considered for analysis. The following AP features were evaluated: resting potential $(V_{{\rm m}_{\rm rest}})$, action potential amplitude (APA), maximum upstroke velocity $(\hat{\dot{V}}_{\rm m})$ and AP duration at 90 % of repolarization (APD_90_). APD_90_ was calculated as the difference between the activation time and the repolarization time. The activation time was determined as the instant of $\hat{\dot{V}}_m$ of the AP. The repolarization time was calculated as the time which the AP waveform crossed a level corresponding to 90 % of repolarization to the resting potential. Conduction velocity (CV) along the muscle was obtained by dividing the distance between two locations (see Fig. 3a) by the difference in arrival times.

Extracellular signals ϕ_e_ at the observation point (CP) were taken for analysis (Fig. 3). The distance of 60 μm to the surface was chosen to match new contact recording systems for extracellular potentials [16]. Other conduction parameters based on extracellular potentials, such as the amplitude $\phi_{{\rm e}_{\rm pp}}$ of the biphasic signal ϕ_e_ calculated as the difference between its positive and negative peaks, the time difference between these two peaks *T*_PP_ and the negative peak of the time derivative $\hat{\dot{\phi}}_e$ were evaluated. Figure 3 summarizes all features which were used to characterize both *V*_m_ and ϕ_e_ dynamics.

### Computational aspects

The bidomain equations were solved using the Cardiac Arrhythmia Research package CARP. Details of the underlying numerical methods have been described elsewhere [28, 29]. Simulation data were stored using a 100 kHz sampling frequency.

## Results

### Effects of ischemia on impulse propagation in a strand

The impact on impulse propagation of the three main components of ischemia at the cellular scale, i.e., hyperkalemia, acidosis and hypoxia, as well as reduced coupling secondary to dephosphorylation of gap junctions at the tissue scale was studied using a numerical model of a cardiac strand. To assess the relative influence of the individual contributing factors to overall ischemia, computer simulations were performed, where the impact of each factor was studied first in separation before combining all of them to form an ischemic strand of tissue.

**Fig. 4:**
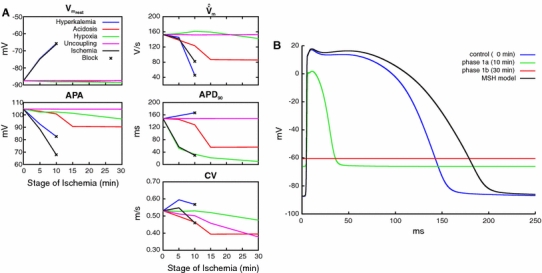
Effects of hyperkalemia, acidosis, hypoxia, cell uncoupling and ischemia (combination of all four components) during impulse propagation on** a** AP properties and CV as well as on** b** AP waveform. Resting potential $(V_{{\rm m}_{\rm rest}}),$ maximum upstroke velocity ($\hat{\dot{V}}_m$), amplitude of transmembrane AP (*APA*), APD at 90 % of repolarization (APD_90_) and conduction velocity (*CV*)

Figure 4a reveals how each component affects AP features and CV. Elevated [K^+^]_e_ has the most prominent influence on $V_{{\rm m}_{\rm rest}}, \hat{\dot{V}}_{\rm m},$ APA and CV. CV increased initially with [K^+^]_e_ (from 5.4 mmol/l at 0 min to 8.9 mmol/l at 5 min, see Table 1), but this trend reversed as hyperkalemia deteriorated until conduction block occurred at [K^+^]_e_ > 8.9 mmol/l, i.e., after 10 min. APA and $\hat{\dot{V}}_m$ monotonically decreased whereas APD_90_ was prolonged up to 12.6 % before conduction block. In contrast, acidosis led to a monotonic decrease in $\hat{\dot{V}}_{\rm m},$ APA, APD_90_ and CV over the first 15 min, while remaining constant afterwards. Hypoxia was the major determinant of APD shortening (from 148 ms under control conditions to 10 ms at t = 30 min), while its impact on all other metrics was rather moderate. Finally, as expected on theoretical grounds, reduced coupling had no influence on AP features, but slowed down CV.

Figure 4b shows simulated APs obtained after combining all contributing factors to model overall ischemia. The AP shape as generated by the original MSH model is shown to serve as a reference. Note that even during normoxic intracellular ATP and ADP levels (6.8 mmol/l and 15 μmol/l, respectively), the augmentation of the MSH model with an *I*_K,ATP_ channel added a non-zero outward K^+^ current which shortened APD_90_ to 80 % of its nominal value. Changes in AP morphology as induced by the progression of ischemia are summarized in Fig. 4a. $V_{{\rm m}_{\rm rest}}$ was elevated while $\hat{\dot{V}}_{\rm m},$ APA and APD_90_ were monotonically reduced until block. The elevation of $V_{{\rm m}_{\rm rest}}$ was determined solely by hyperkalemia (the blue trace related to hyperkalemia overlaps with the black trace related to ischemia). APA was reduced to a larger extent due to the combined effects of hyperkalemia, acidosis and hypoxia. Shortening of APD_90_ was mainly governed by the hypoxia-driven increase in *I*_K,ATP_. Hyperkalemia led to conduction block after 10 min, no APs could be elicited at 15 min (not shown) and 30 min (phase 1b).

### Effects of ischemia-induced heterogeneity on impulse propagation and AP characteristics in a 3D preparation

**Fig. 5:**
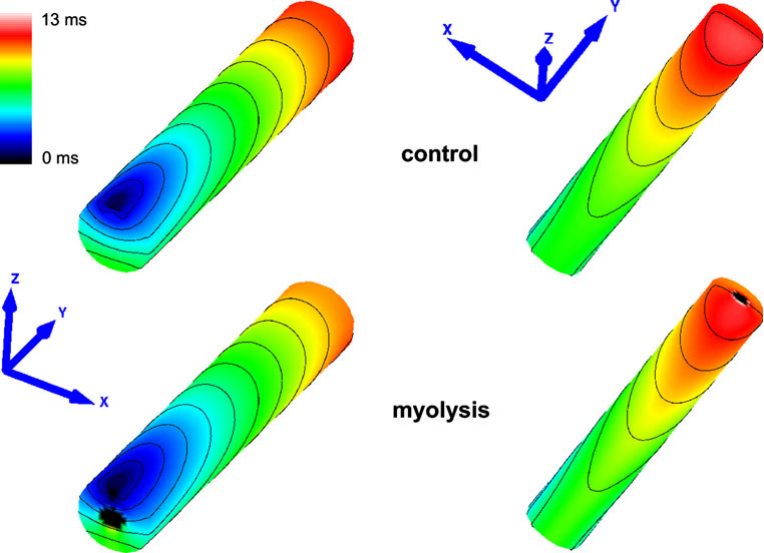
Activation sequences in the control and myolysis models (viewing* upper* and* back* sides of muscle). Activation started at the* left top cusp* (*blue*) and finished after 11.5 ms (control) and 12.9 ms (myolysis) at the right basal cusp (*red*)

**Fig. 6:**
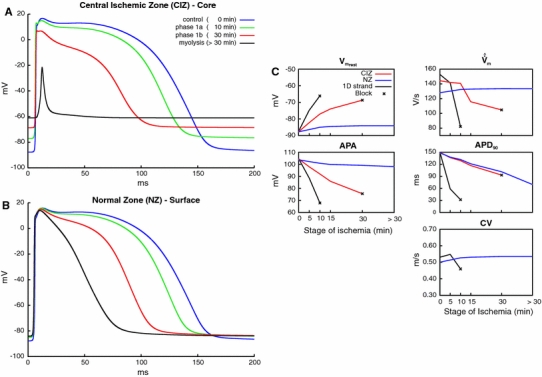
Results obtained with all 3D models. Representative APs** a** in the core (CIZ) and** b** in the surface layer (NZ) for varying states of progression of ischemia in the CIZ: control, ischemia phase 1a, phase 1b and myolysis.** c** Variation of AP properties in CIZ and NZ as a function of ischemia progression (see Tables 1, 2). Results related to the 1D strand are shown for the purpose of comparison

A 3D model of a thin-walled cardiac muscle was used to study the effects of ischemia-induced radial heterogeneity on activation sequence and AP characteristics. Figure 5 presents the propagation pattern in control as well as in myolysis (worst case scenario) and Fig. 6 shows APs in both the CIZ and the NZ of the muscle. Unlike with the homogeneous 1D strand where functional gradients were absent, electrotonic interactions due to intrinsic differences in AP morphology between the CIZ and viable NZ act to modulate the intrinsic cellular dynamics. Comparing Figs. 6a and 4b demonstrates that main qualitative differences were observed in case of phase 1B and myolysis. Unlike in the case of the 1D strand, *V*_m_ in the CIZ always departed from the resting potential, even in the case of myolysis, driven by electrotonic interactions between NZ and CIZ. Conduction within the CIZ was possible during phase 1B and failed only in the case of myolysis (>30 min) where ${\varvec{\sigma}}_{\rm i}$ in the whole core (CIZ and BZ) was set to 10 % of the control model. CV in the NZ increased by 5.2 % during phase 1a, but remained constant during more advanced stages of ischemia. Figure 5 reveals that despite the fact that propagation fails in the CIZ during myolysis, the overall activation sequence at the surface is comparable to the control.

At the same time, the presence of a CIZ adjacent to the viable NZ had a very minor impact on all AP characteristics, except APD_90_. Changes in $V_{{\rm m}_{\rm rest}},$ APA and $\hat{\dot{V}}_m$ were detectable, but remained small (<5.5 %). The most prominent effect was a significant shortening of APD_90_ due to diffusive repolarization currents. APD_90_ shortened from 148.6 ms (control) to 131.9 ms, 88.8 % (phase 1a), 101.4 ms, 68.2 % (phase 1b) and 69.8 ms, 47.0 % (myolysis), respectively. That is, in the most extreme case of a myolytic core APD_90_ in the viable NZ decreased to 47 % of those values observed in a muscle where the entire cross section remains viable. All changes in AP characteristics over time in NZ and CIZ under electrotonic coupling are shown in Fig. 6c.

### Effect of ischemia on extracellular depolarization signals recorded at the surface

**Fig. 7:**
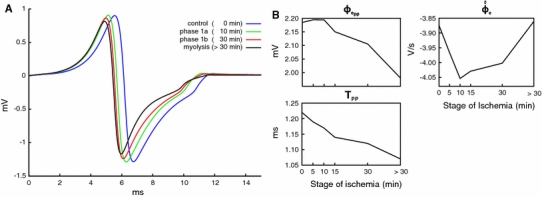
Signals recorded close to the surface of the preparation at observation the point CP (see Fig. 1).** a** Extracellular potentials recorded at different stages of ischemia: control, ischemia phase 1a, phase 1b and myolysis.** b** Variation of ϕ_e_-derived metrics during ischemia progression. Amplitude of the signal $(\phi_{{\rm e}_{\rm pp}}),$ negative peak of the time derivative $\hat{\dot{\phi}}_e$ and time difference between the negative and positive peaks *T*_pp_

Extracellular signals ϕ_e_ recorded at the observation site CP are shown in Fig. 7a for different stages during the progression of ischemia. Note that the shape of the depolarization signals are very similar. As ischemia progresses, minor delays relative to the control case occur due to the minor conduction slowing in the NZ secondary to the drag effect of the CIZ. Further, minor differences are observed between 8 and 12 ms due to border effects which arise when the wavefront collides with the end of the specimen. Figure 7b reveals that all conduction parameters based on ϕ_e_ are marginally affected by the ischemic core. Comparing control with phase 1b and myolysis we observe that $\phi_{{\rm e}_{\rm pp}}$ is decreased by 3.6 and 9.4 %, whereas the peak-to-peak interval *T*_PP_ is shortened by 8.2 and 12.3 %, respectively. The maximum negative rate of rise $\hat{\dot{\phi}}_e$ showed a non-monotonic behavior, being more negative by 3.3 % during phase 1b, but returning to the control value during myolysis, where the CIZ was almost fully decoupled.

## Discussion

Since the 1980s, superfusion techniques have been used extensively as a method to supply oxygen and nutrients to thin-walled cardiac tissue samples such as pectinate and papillary muscles [15, 21, 23, 26]. In recent years, superfusion has gained relevance again in experimental studies that involve large tissue samples [17, 22, 40]. Such thin-walled samples, although virtually 1D (trabeculae, pectinate or papillary muscles) or 2D (atria), are effectively 3D structures which is why gradients in metabolic state build up over the cross section of a muscle, unless more elaborate perfusion techniques are applied [7, 19, 42]. Over time, the core of the muscle becomes ischemic whereas superficial layers remain perfectly viable. It remains unclear as to which degree electrophysiological recordings from such preparations are affected. Computer simulations suggests themselves quite naturally as a modality for investigating these effects, since electrophysiological parameters in the depth of such samples cannot be observed at all, or with very limited spatio-temporal resolution, with experimental techniques alone. Previously, the impact of ischemia progression on impulse propagation in such thin-walled superfused preparations has been studied using 1D computer models [38], however, effects of radial heterogeneity, as they arise in 3D, have not been considered yet. In this study, a 3D computational model was developed to characterize the effects of superfusion-induced radial heterogeneity on AP morphology and impulse propagation, as well as to assess the relative contributions of superficial and core layers to extracellular depolarization signals measured in the volume conductor close to the tissue surface. Although the main focus of the study is on the impact on depolarization signals and propagation, repolarization metrics such as changes in APD are reported as well, since these are of relevance when interpreting our results in the context of larger tissues samples when used for studying arrhythmogenesis or defibrillation.

### Effects of ischemia in a 1D strand

The recent MSH model of a rabbit ventricular myocyte was used and augmented following previously reported approaches [30, 34, 39, 45] to account for the three main effects of ischemia at the cellular level: hyperkalemia, acidosis and hypoxia [3, 30, 34, 39, 45]. 1D simulations were performed to ascertain that predictions are in line with previous studies: (1) Ischemia-induced changes are qualitatively and quantitatively reproduced for all stages of ischemia progression (Fig. 4). (2) The biphasic relationship between CV and [K^+^]_e_, referred to as “supernormal conduction” shown in Fig. 4a [38], was observed. (3) As previously reported [38], conduction block occurred at [K^+^]_e_ > 8.9 mmol/l (10 min). (4) Augmentation with an ATP-sensitive K^+^ channel shortened APD_90_ by 20 % under normoxic conditions, in agreement with the previous studies where decreases of 17 % [45] and 12 % [13] were reported. (5) Under normoxic conditions, AP characteristics matched up very well with other simulation studies [34, 38, 39, 45] and fell within the range of experimental data obtained from perfused rabbit papillary muscles [7, 19, 20, 42, 43]. (6) Progressive uncoupling and morphological changes in the interstitial matrix, as reflected by changes in extra- and intracellular conductivities in the model, did not influence any AP properties. The decrease in ${\varvec{\sigma}}_e$ during the first 10 min led to a small decrease in CV and later after >15 min to disturbances of impulse conduction, consistent with experimental reports [20].

### Effects of ischemia in a 3D preparation

In 3D, the intrinsic cellular dynamics is modulated by electrotonic crosstalk between cells of different electrophysiological states. This caused APD to be noticeably shorter in the viable superficial NZ due to the presence of additional diffusive repolarization currents, as imposed by cells in the CIZ where APD is much shorter or tissue even remains unexcited. Conversely, APDs in the CIZ are longer and APs are elicited even in those cases where propagation block occurred in 1D. These electrotonic influences are governed by the transverse space constant, λ_t_, which is at the order of ≈100 μm. That is, electrotonic crosstalk occurs over the entire cross section of the preparation, assuming a radius of influence of 5 × λ_t_. Therefore, intrinsic differences between CIZ and viable NZ were smoothed out, resulting in ischemic effects which are qualitatively similar to 1D, but significantly less pronounced.

Major global physiological differences arose mainly at more progressive stages of ischemia. Unlike in 1D where conduction block occurred at the onset of phase 1b (15 min), propagation in 3D did not even fail in the late phase 1b (30 min). Conduction block occurred only during myolysis (>30 min) where ${\varvec{\sigma}}_i$ in the core was reduced to 10 % of the control values. Further, as ischemia progressed, the overall activation sequence started to alter. These changes were moderate until phase 1b, affecting mainly the shape of the depolarization wavefront in the CIZ. In the extreme scenario of myolysis propagation failed in the CIZ, which caused the activation wavefront to propagate around the myolytic core. This led to collisions at the bottom of the muscle (see Fig. 5). The activation sequence at the top of the muscle remained virtually unchanged though, only minor difference were observed. At the surface, CV is slightly faster in myolysis, as shown in Figs. 5 and 6c in the CV panel, because the myolitic core is decoupled from the the healthy tissue (${\varvec{\sigma}}_i$ was reduced to 10  %). Thus, unlike the control condition, the core does not represent a load for the activated cells at the surface. In terms of total activation time, in myolysis this took slightly longer than in the control case with 12.9 versus 11.5 ms, respectively. This is caused by cells within the BZ, which are still able to conduct a very impaired AP due to electrotonic interactions between NZ and CIZ. That is, the latest activating cells are located in the distal end of the preparation in the BZ (not shown).

### Extracellular depolarization signals

Despite major electrophysiological changes throughout the muscle, recordings of extracellular depolarization signals close to the surface remained surprisingly unaffected. For instance, during phase 1b extracellular potentials ϕ_e_ at CP had their *T*_PP_ shortened by 8.2 % and changes in $\phi_{{\rm e}_{\rm pp}}$ as well as in $\hat{\dot{\phi}}_e$ were even less significant (<4 %), as shown in Fig. 7b. In the extreme case of myolysis where conduction was blocked in the CIZ, the effect on extracellular depolarization signals was very minor, with a 9.4 % decrease in $\phi_{{\rm e}_{\rm pp}}$ and a shortening of *T*_PP_ by 12.3 % compared to control. Minor changes in arrival times arose due to changes in CV.

These fairly moderate changes can be attributed to the fact that sources in the superficial NZ are closer to the recording site and thus carry a more significant weight in determining the extracellular potential field. Contributions of sources to ϕ_e_ are weighted by a 1/*r* relationship where *r* is the distance between recording site and source point [27]. Hence, for the given setup sources in the NZ carry roughly ten times the weight of sources located in the more distant CIZ. The temporal derivative $\dot{\phi}_{\rm e}$ was even less affected since weighting of sources is governed by the function 1/*r* ^2^, giving sources in the NZ ∼87 times the weight of sources in the CIZ. Further, the sources carrying most of the weight are located in the NZ which is least affected. In the NZ $V_{{\rm m}_{\rm rest}}, \hat{\dot{V}}_m$ and APA were barely affected (see Fig. 6), only APD_90_ was significantly altered there, with reductions of 32 and 53 % during phase 1b and myolysis, respectively. However, changes in APD are not reflected at all in extracellularly recorded depolarization signals.

### Limitations of the model

A 3D computational model of a superfused thin-walled cardiac muscle was developed, using a cylindrical geometry with a diameter of 1.0 mm to match experimental set-ups [19, 42, 43]. This choice of diameter is a key factor which strongly influences the electrophysiology of the whole preparation, in particular, the ratio between viable and ischemic tissue volume. Nonetheless, in this study, the diameter was not a model parameter and kept fixed throughout the study. A further important factor is the width of viable NZ and BZ which depends on numerous factors such as the rate of cellular consumption, the diameter of the muscle or the pressure in the external solution. Even when the superfusate is well oxygenated, the CIZ may be still deprived of an adequate oxygen supply if oxygen use is high, diffusion distances are excessively large, or both [26]. Experiments with rabbit myocardium, where perfusion and superfusion were combined, demonstrated that cells within 130 μm remain viable, whereas cells up to 650 μm are relatively little affected by ischemia phase 1a [46]. In the same study, transmural extracellular gradients of K^+^, pH and myocardial energy metabolism were also shown to build up, depressing APs markedly in layers deeper than 600 μm in phase 1b. Such transmural inhomogeneities in the ischemic heart have been further investigated in other experimental work as well [8, 37]. Early modeling and experimental studies suggested that the critical diameter is around 0.6–0.8 mm at 37 °C, where, if exceeded, an anoxic core is formed [2, 9, 26]. In our model, the widths of the NZ and BZ were kept fixed as 250 and 125 μm, respectively, which is consistent with experimental reports [1, 46], in the range of 130–300 μm. Moreover, the width of NZ and BZ is also known to be different for the three main components of ischemia [35]. However, we opted for a simpler approach where the same width of BZ for hyperkalemia, acidosis and hypoxia was used and all parameters in Tables 1 and 2 were linearly interpolated between NZ and CIZ [3, 18].

A further limitation is the short length and simplified geometry of the model. While this setup approximates traditional experimental setups fairly well where isolated papillary muscles or trabeculae are used, this is not the case with more recent studies [17, 22, 40] where electrical activity is globally mapped over larger preparations such as the atria (as shown in Fig. 1). Nonetheless, the findings reported in this study can be extrapolated to such larger superfused preparation, considering that the thickness of viable layer is the quantity of primary importance. Our results suggest that activation sequences and extracellular potentials during the depolarization phase will remain largely unaffected in larger preparations, however, particular caution is advised when analyzing data which also involve the repolarization phase, as it is the case when studying the formation of arrhythmias.

Although these choices may affect the findings presented in this study quantitatively, we believe that qualitatively our findings are robust and do not depend on these choices. With regard to measurements of extracellular depolarization signals, this view is supported by the observation that even in the worst case scenario of a myolytic core the influence on ϕ_e_ was virtually negligible.

### Conclusion

Despite the numerous significant changes in electrophysiological properties, the extracellular potential field close to the surface of superfused preparations remains virtually unaffected. Depending on the question under study, superfused preparations have to be used with care, particularly with regard to repolarization. Despite some limitations, simulation results suggest that superfusion techniques are perfectly adequate when studying impulse propagation on thin-walled tissue preparations.

## References

[1] de Bakker JM, van Capelle FJ, Janse MJ, Tasseron S, Vermeulen JT, de Jonge N, Lahpor JR (1993). Slow conduction in the infarcted human heart. ’Zigzag’ course of activation. Circulation.

[2] Barclay CJ (2005). Modelling diffusive O(2) supply to isolated preparations of mammalian skeletal and cardiac muscle. J Muscle Res Cell Motil.

[3] Bernus O, Zemlin CW, Zaritsky RM, Mironov SF, Pertsov AM (2005). Alternating conduction in the ischaemic border zone as precursor of reentrant arrhythmias: a simulation study. Europace.

[4] Bersohn MM (1995). Sodium pump inhibition in sarcolemma from ischemic hearts. J Mol Cell Cardiol.

[5] Bussek A, Wettwer E, Christ T, Lohmann H, Camelliti P, Ravens U (2009). Tissue slices from adult mammalian hearts as a model for pharmacological drug testing. Cell Physiol Biochem.

[6] Carmeliet E (1999). Cardiac ionic currents and acute ischemia: from channels to arrhythmias. Physiol Rev.

[7] Cascio WE, Yan GX, Kléber AG (1990). Passive electrical properties, mechanical activity, and extracellular potassium in arterially perfused and ischemic rabbit ventricular muscle. Effects of calcium entry blockade or hypocalcemia. Circ Res.

[8] Coronel R, Fiolet JW, Wilms-Schopman FJ, Schaapherder AF, Johnson TA, Gettes LS, Janse MJ (1988). Distribution of extracellular potassium and its relation to electrophysiologic changes during acute myocardial ischemia in the isolated perfused porcine heart. Circulation.

[9] Daut J, Elzinga G (1988). Heat production of quiescent ventricular trabeculae isolated from guinea-pig heart. J Physiol.

[10] De Groot JR, Coronel R (2004). Acute ischemia-induced gap junctional uncoupling and arrhythmogenesis. Cardiovasc Res.

[11] Doering AE, Lederer WJ (1993). The mechanism by which cytoplasmic protons inhibit the sodium–calcium exchanger in guinea-pig heart cells. J Physiol.

[12] Dos Santos RW, Otaviano Campos F, Neumann Ciuffo L, Nygren A, Giles W, Koch H (2006). ATX-II effects on the apparent location of M cells in a computational model of a human left ventricular wedge. J Cardiovasc Electrophysiol.

[13] Ferrero JM, Saiz J, Ferrero JM, Thakor NV (1996). Simulation of action potentials from metabolically impaired cardiac myocytes. Role of ATP-sensitive K+ current. Circ Res.

[14] Geselowitz DB, Miller WT (1983). A bidomain model for anisotropic cardiac muscle. Ann Biomed Eng.

[15] Guo ZG, Levi R, Aaronson LM, Gay WA (1983). The isolated human pectinate muscle: a reliable preparation of human cardiac tissue. J Pharmacol Methods.

[16] Hofer E, Keplinger F, Thurner T, Wiener T, Sanchez-Quintana D, Climent V, Plank G (2006). A new floating sensor array to detect electric near fields of beating heart preparations. Biosens Bioelectron.

[17] Hucker WJ, Sharma V, Nikolski VP, Efimov IR (2007). Atrioventricular conduction with and without AV nodal delay: two pathways to the bundle of H is in the rabbit heart. Am J Physiol Heart Circ Physiol.

[18] Jie X, Trayanova N (2010). Mechanisms for initiation of reentry in acute regional ischemia phase 1B. Heart Rhythm.

[19] Kléber AG, Riegger CB (1987). Electrical constants of arterially perfused rabbit papillary muscle. J Physiol.

[20] Kléber AG, Riegger CB, Janse MJ (1987). Electrical uncoupling and increase of extracellular resistance after induction of ischemia in isolated, arterially perfused rabbit papillary muscle. Circ Res.

[21] Kockskamper J, von Lewinski D, Khafaga M, Elgner A, Grimm M, Eschenhagen T, Gottlieb PA, Sachs F, Pieske B (2008). The slow force response to stretch in atrial and ventricular myocardium from human heart: functional relevance and subcellular mechanisms. Prog Biophys Mol Biol.

[22] Lau DH, Mackenzie L, Shipp NJ, Kuklik P, Dimitri H, Lobb BL, Alasady M, Lim HS, Kelly DR, Brooks AG, Saint DA, Sanders P (2010). Feasibility of high-density electrophysiological study using multiple-electrode array in isolated small animal atria. Clin Exp Pharmacol Physiol.

[23] Lemoine S, Puddu PE, Durand C, Lepage O, Babatasi G, Ivascau C, Massetti M, Gerard JL, Hanouz JL (2010). Signaling pathways involved in postconditioning-induced cardioprotection of human myocardium, in vitro. Exp Biol Med (Maywood).

[24] Mahajan A, Shiferaw Y, Sato D, Baher A, Olcese R, Xie LH, Yang MJ, Chen PS, Restrepo JG, Karma A, Garfinkel A, Qu Z, Weiss JN (2008). A rabbit ventricular action potential model replicating cardiac dynamics at rapid heart rates. Biophys J.

[25] Malloy CR, Buster DC, Castro MM, Geraldes CF, Jeffrey FM, Sherry AD (1990). Influence of global ischemia on intracellular sodium in the perfused rat heart. Magn Reson Med.

[26] Paradise NF, Schmitter JL, Surmitis JM (1981). Criteria for adequate oxygenation of isometric kitten papillary muscle. Am J Physiol.

[27] Plank G, Hofer E (2003). Use of cardiac electric near-field measurements to determine activation times. Ann Biomed Eng.

[28] Plank G, Liebmann M, Weberdos Santos R, Vigmond EJ, Haase G (2007). Algebraic multigrid preconditioner for the cardiac bidomain model. IEEE Trans Biomed Eng.

[29] Plank G, Zhou L, Greenstein JL, Cortassa S, Winslow RL, O’Rourke B, Trayanova NA (2008). From mitochondrial ion channels to arrhythmias in the heart: computational techniques to bridge the spatio-temporal scales. Philos Transact A Math Phys Eng Sci.

[30] Pollard AE, Cascio WE, Fast VG, Knisley SB (2002). Modulation of triggered activity by uncoupling in the ischemic border. A model study with phase 1b-like conditions. Cardiovasc Res.

[31] Prassl AJ, Kickinger F, Ahammer H, Grau V, Schneider JE, Hofer E, Vigmond EJ, Trayanova NA, Plank G (2009). Automatically generated, anatomically accurate meshes for cardiac electrophysiology problems. IEEE Trans Biomed Eng.

[32] Raman S, Kelley MA, Janssen PM (2006). Effect of muscle dimensions on trabecular contractile performance under physiological conditions. Pflugers Arch.

[33] Riegger CB, Alperovich G, Kléber AG (1989). Effect of oxygen withdrawal on active and passive electrical properties of arterially perfused rabbit ventricular muscle. Circ Res.

[34] Rodriguez B, Ferrero JM, Trenor B (2002). Mechanistic investigation of extracellular K+ accumulation during acute myocardial ischemia: a simulation study. Am J Physiol Heart Circ Physiol.

[35] Rodriguez B, Trayanova N, Noble D (2006). Modeling cardiac ischemia. Ann N Y Acad Sci.

[36] Sachse FB, Steadman BW, B Bridge JH, Punske BB, Taccardi B (2004). Conduction velocity in myocardium modulated by strain: measurement instrumentation and initial results. Conf Proc IEEE Eng Med Biol Soc.

[37] Schaapherder AF, Schumacher CA, Coronel R, Fiolet JW (1990). Transmural inhomogeneity of extracellular [K+] and pH and myocardial energy metabolism in the isolated rat heart during acute global ischemia; dependence on gaseous environment. Basic Res Cardiol.

[38] Shaw RM, Rudy Y (1997). Electrophysiologic effects of acute myocardial ischemia. A mechanistic investigation of action potential conduction and conduction failure. Circ Res.

[39] Shaw RM, Rudy Y (1997). Electrophysiologic effects of acute myocardial ischemia: a theoretical study of altered cell excitability and action potential duration. Cardiovasc Res.

[40] Sicouri S, Gianetti B, Zygmunt AC, Cordeiro JM, Antzelevitch C (2011). Antiarrhythmic effects of simvastatin in canine pulmonary vein sleeve preparations. J Am Coll Cardiol.

[41] Stinstra JG, Shome S, Hopenfeld B, MacLeod RS (2005). Modelling passive cardiac conductivity during ischaemia. Med Biol Eng Comput.

[42] Tan HL, Janse MJ (1994). Contribution of mechanical activity and electrical activity to cellular electrical uncoupling in ischemic rabbit papillary muscle. J Mol Cell Cardiol.

[43] Tan HL, Mazon P, Verberne HJ, Sleeswijk ME, Coronel R, Opthof T, Janse MJ (1993). Ischaemic preconditioning delays ischaemia induced cellular electrical uncoupling in rabbit myocardium by activation of ATP sensitive potassium channels. Cardiovasc Res.

[44] Tranum-Jensen J, Janse MJ, Fiolet WT, Krieger WJ, D’Alnoncourt CN, Durrer D (1981). Tissue osmolality, cell swelling, and reperfusion in acute regional myocardial ischemia in the isolated porcine heart. Circ Res.

[45] Weiss DL, Ifland M, Sachse FB, Seemann G, Dossel O (2009). Modeling of cardiac ischemia in human myocytes and tissue including spatiotemporal electrophysiological variations. Biomed Tech (Berl).

[46] Wilensky RL, Tranum-Jensen J, Coronel R, Wilde AA, Fiolet JW, Janse MJ (1986). The subendocardial border zone during acute ischemia of the rabbit heart: an electrophysiologic, metabolic, and morphologic correlative study. Circulation.

[47] Zimmer HG (1998). The isolated perfused heart and its pioneers. News Physiol Sci.

